# Supplementary figures and images for: Aspirin modulates production of pro-inflammatory and pro-resolving mediators in endothelial cells
Source: PLoS One. 2023 Apr 25;18(4):e0283163. doi: 10.1371/journal.pone.0283163 (PMC10128936; doi:10.1371/journal.pone.0283163)

## Slide 1
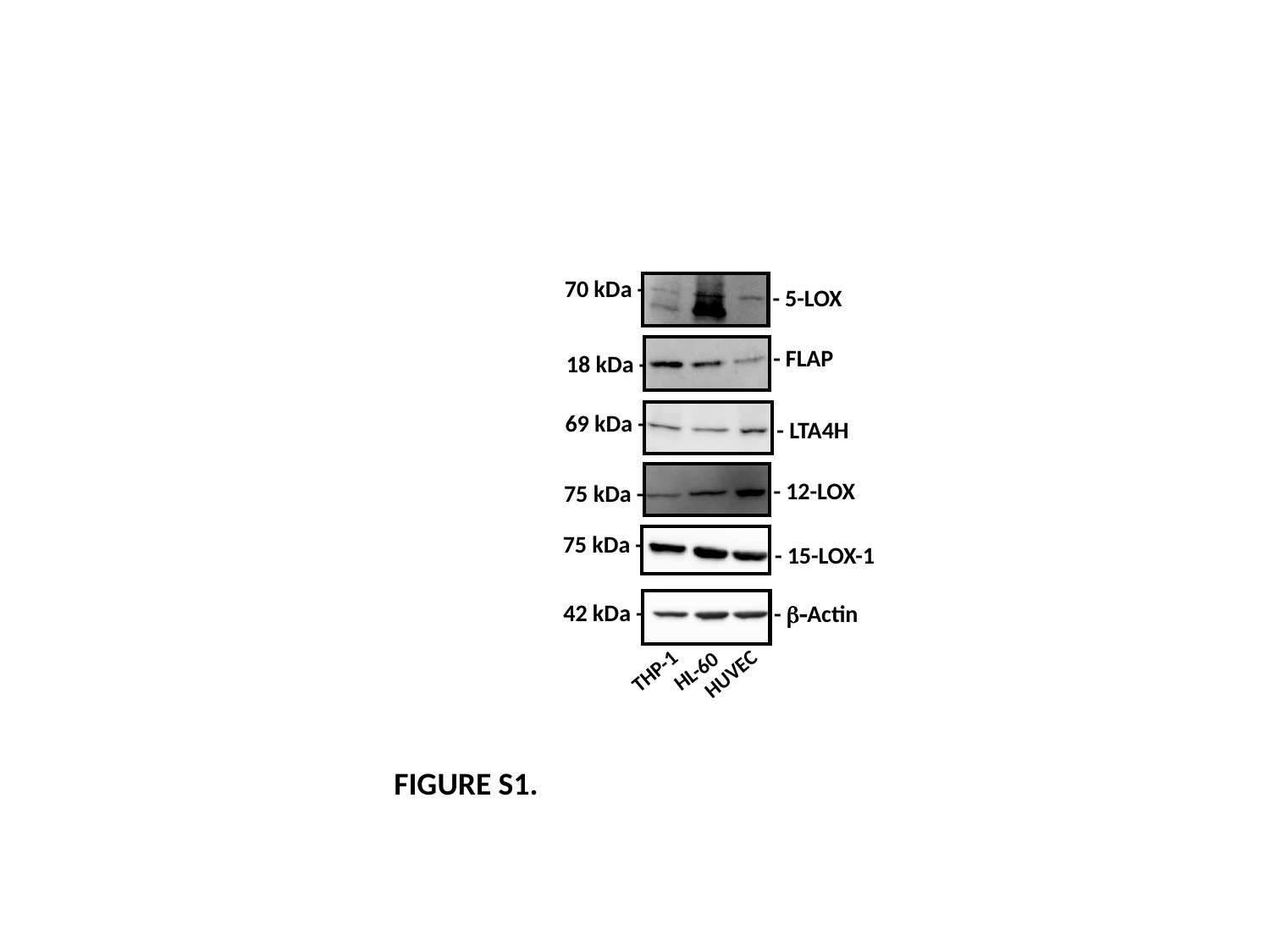

70 kDa -
- 5-LOX
- FLAP
- LTA4H
- 12-LOX
- 15-LOX-1
- b-Actin
18 kDa -
69 kDa -
75 kDa -
75 kDa -
42 kDa -
THP-1
HL-60
HUVEC
FIGURE S1.

Supplement: S1 Fig — (5-lipoxygenase, 5-LOX; 5-lipoxygenase-activating protein, FLAP; leukotriene A4 hydrolase, LTA4H; 12-lipoxygenase, 12-LOX; 15-lipoxygenase-1, 15-LOX-1; β−Actin). Lane 1, PMA-differentiated THP-1 cells; lane 2, DMSO-differentiated HL-60 cells; lane 3, unstimulated HUVEC. Cell extracts (30 μg/lane) were fractionated on 4–20% SDS-PAGE, transferred to nitrocellulose and probed with antibodies (see S1 Table). (PPTX) [file pone.0283163.s001.pptx]
