# Supplementary material for: Aspirin modulates production of pro-inflammatory and pro-resolving mediators in endothelial cells
Source: PLoS One. 2023 Apr 25;18(4):e0283163. doi: 10.1371/journal.pone.0283163 (PMC10128936; doi:10.1371/journal.pone.0283163)
Supplement: S1 Table — Polyclonal or monoclonal antibodies were used at the indicated dilutions. Where possible, positive controls (THP-1, HL-60 cell lysates for 5-LOX, 15-LOX, LTA4H and FLAP proteins) or recombinant proteins for COX-1 and COX-2. In addition, negative controls also consisted of pre-absorption of primary antibodies with blocking peptides where available. (DOCX) [file pone.0283163.s002.docx]

| **Protein Target** | **M*_r_* (kDa)** | **Species, Clonality** | **Dilution** | **Source** | **Cat #** |
| --- | --- | --- | --- | --- | --- |
| Cyclooxygenase-1, COX-1 | 72 | Rabbit, Monoclonal | 1:1000 | ProteinTech | #13393-1-AP |
| Cyclooxygenase-2, COX-2 | 72 | Mouse, Monoclonal | 1:1000 | Invitrogen | #MA5-14568 |
| Prostacyclin Synthase, PGIS | 55 | Rabbit, Polyclonal | 1:500 | Invitrogen | #PA5-102017 |
| Microsomal, Prostaglandin E Synthase-1, mPGES-1 | 21 | Rabbit, Polyclonal | 1:1000 | Invitrogen | #PA5-60916 |
| 5-Lipoxygenase, 5-LOX | 70 | Rabbit, Polyclonal | 1:1000 | Abcam | #ab169755 |
| 12-Lipoxygenase, 12-LOX | 75 | Mouse, Monoclonal | 1:1000 | Abcam | #ab211506 |
| 15-Lipoxygenase, 15-LOX | 75 | Rabbit, Polyclonal | 1:1000 | Abcam | #ab119774 |
| 5-Lipoxygenase-activating Protein, FLAP | 18 | Rabbit, Monoclonal | 1:1000 | Invitrogen | #MA5-37933 |
| Leukotriene A Hydrolase, LTA4H | 69 | Rabbit, Polyclonal | 1:1000 | Abcam | #ab133512 |
| β−Actin | 42 | Mouse, Monoclonal | 1:10000 | Sigma-Aldrich | #A1978 |

**Table S1. Antibodies.** Polyclonal or monoclonal antibodies were used at the indicated dilutions. Where possible, positive

controls (THP-1, HL-60 cell lysates for 5-LOX, 15-LOX, LTA4H and FLAP proteins) or recombinant proteins for COX-1 and COX-2.

In addition, negative controls also consisted of pre-absorption of primary antibodies with blocking peptides where available.
