# Supplementary material for: Aspirin modulates production of pro-inflammatory and pro-resolving mediators in endothelial cells
Source: PLoS One. 2023 Apr 25;18(4):e0283163. doi: 10.1371/journal.pone.0283163 (PMC10128936; doi:10.1371/journal.pone.0283163)
Supplement: S2 Table — All qRT-PCR experiments were conducted using Taqman™ chemistry and data were analyzed using the 2-ΔΔCt method. (DOCX) [file pone.0283163.s003.docx]

| Gene Name | Gene Symbol | *Taqman* Assay ID No. | Amplicon Size (bp) |
| --- | --- | --- | --- |
| Prostaglandin Endoperoxide Synthase-1, Cyclooxygenase-1 | PTGS-1, COX-1 | Hs00377726_m1 | 60 |
| Prostaglandin Endoperoxide Synthase-2, Cyclooxygenase-2 | PTGS-2, COX-2 | Hs00153133_m1 | 75 |
| Prostacyclin Synthase | PTGIS, PGIS | Hs00168766_m1 | 106 |
| Microsomal Prostaglandin E Synthase-1 | PTGES, mPGES-1 | Hs00610420_m1 | 68 |
| 5-Lipoxygenase | ALOX5 | Hs00167536_m1 | 57 |
| 12-Lipoxygenase | ALOX12 | Hs00167524_m1 | 59 |
| 15-Lipoxygenase | ALOX15 | Hs00993765_g1 | 64 |
| Leukotriene A_4_ Hydrolase | LTA4H | Hs01075871_m1 | 77 |
| 5-Lipoxygenase-activating Protein | ALOX5AP, FLAP | Hs00970921_m1 | 80 |
| Glyceraldehyde 3-phosphate Dehydrogenase | GAPDH | Hs02786624_g1 | 157 |

**Table S2. PCR Primer Sets.** All qRT-PCR experiments were conducted using Taqman™ chemistry and data were

analyzed using the 2^-ΔΔ^*^Ct^* method.
