# Supplementary material for: Aspirin modulates production of pro-inflammatory and pro-resolving mediators in endothelial cells
Source: PLoS One. 2023 Apr 25;18(4):e0283163. doi: 10.1371/journal.pone.0283163 (PMC10128936; doi:10.1371/journal.pone.0283163)
Supplement: S3 Table — All analytes were below the lower limit of detection. Duplicate samples were analyzed in two separate experiments. (DOCX) [file pone.0283163.s004.docx]

| **Lipid Analyte** | **%Bound** | **Conc. (pg/ml)** |
| --- | --- | --- |
| 6-ketoPGF_1α_ | 105.4 | <1.6 |
| PGE_2_ | 103.8 | <15.6 |
| LTB_4_ | 101.8 | <15.6 |
| LXA_4_ | 102.7 | <8.19 |
| 15-epi-LXA_4_ | 98.4 | <3.3 |

**Table S3.** M199 media supplemented with 0.5% charcoal-stripped serum (0.5% FCS) and 5% Endothelial Cell Growth Supplement (5% ECGS) not exposed to cells was analyzed for lipid analytes listed in the table. All analytes were below the lower limit of detection. Duplicate samples were analyzed in two separate experiments.
