# Supplementary material for: Aspirin modulates production of pro-inflammatory and pro-resolving mediators in endothelial cells
Source: PLoS One. 2023 Apr 25;18(4):e0283163. doi: 10.1371/journal.pone.0283163 (PMC10128936; doi:10.1371/journal.pone.0283163)
Supplement: Attachment — Submitted filename: Responses to Reviewers.docx [file pone.0283163.s005.docx]

**Responses to Reviewers’ Comments:**

**General comments:**

1. **Concern about the specificity of ELISA assays to unequivocally determine the production of specific lipid mediators.** **Response:** We recognize that the gold standard for the measurement of lipids in biological matrices is LC-MS/MS, typically using triple quadrupole mass spectrometry. In the current work, we used highly specific and thoroughly validated ELISAs from Cayman Chemical Co. to measure (6keto PGF_1a_, the stable metabolite of PGI_2_, PGE_2_, LTB_4_, LXA_4_ and 15-epi-LXA_4_ (Aspirin-triggered Lipoxin A4). Each of these solid-phase immunoassays has been used by many authors in high-profile publications. I have had in-depth discussions with the scientific staff at Cayman Chemical regarding any nuances with these assays. Each assay has been extensively tested for cross-reactivity (this information is included in the Supplementation Information included in the revised manuscript. We did not resort to LC-MS/MS in this particular manuscript due to financial limitations. Moreover, the use of 96-well ELISA formats permits the execution of large numbers of biological replicates that can be subjected to statistical testing. It would have been cost-prohibitive to have done the same number of replicates using LC-MS/MS. (My lab is working carefully with the Lipidomics Core facility at Wayne State University in Detroit, MI and with Cayman Chemical Co, Ann Arbor, MI to confirm the authenticity, accuracy, precision, specificity and sensitivity of the ELISA in comparison to conventional LC-MS/MS.). Dr. Gowdy has recently published a paper that demonstrated strong agreement between ELISA and LC-MS/MS values for PGE_2_ in lung tissue specimens (Yeager et al., *Toxicol Sci*, 2021; 183: 170, PMID: 34175951). Finally, we have conducted new experiments using pharmacological inhibitors of key enzymes and proteins in the lipid synthesizing pathways (e.g., 5-lipoxygenase inhibitor, Zileuton; FLAP inhibitor, MK-886) to confirm the results and conclusions in the original manuscript submission.

**2.	15-LO expression should be directed determined in HUVEC and preferably in some fashion that allow directly association of enzyme expression and the cell s containing endothelial markers such as immunofluorescence.** **Response:** The culture system used in this work is HUVEC that are 100% endothelial in nature. There is no evidence of contamination from any other cell type. Thus, although it cannot be concluded that equal expression of all lipid synthesizing enzymes is found in each and every cell in the cultures, nonetheless, it is valid to conclude that whatever enzyme expression is detected (by qRT-PCR and western blotting) is the located in endothelial cells.

**Specific responses to reviewers’ comments:**

1. **Are the authors sure that the immunoassays applied to measure lipid mediators in complex mixtures of cell extract are specific for the indicated lipid mediator. They should verify their results with LC-MS analyses. Current LC-MS assays are more sensitive than the immunoassays. Response:** 1. We are familiar with the attached paper outlining LC-MS/MS methods. We agree that LC-MS/MS continues to be the gold standard for lipidomics. In the current work we made the calculated decision to use ELISA methods in order to conduct many experiments using biological replicates. This would have been cost-prohibitive if LC-MS/MS were the selected method, since each sample costs approximately $100 to prepare and analyze. The ELISA assays used in this work were from Cayman Chemical Co. and have been extensively validated by the company and in-house for specificity and sensitivity. More detail on our in-house validation is presented in the Supplemental Information section of this revised manuscript. I have had extensive discussions with the scientific staff at Cayman Chemical and with Dr. Krishna Rao Maddipati, Director of the Lipidomics Core at Wayne State University regarding the pros and cons of ELISA versus LC-MS/MS. While LC-MS/MS is highly specific in identifying the precise structure of lipids, it is less sensitive than ELISA on a molar basis. Moreover, it is typically quite sensitive the matrix used. In the current work, we did not use cell extracts as implied by the reviewer’s question, rather we collected culture media. The medium was supplemented only with 0.5% charcoal-stripped FBS and endothelial cell growth supplement (from bovine brain extract). We tested the serum and supplement for the lipids in question and they were at or below the limit of detection. Thus, lipids measured in the media represent those produced by HUVEC and not contaminants from the supplements. Finally, the ELISAs for each lipid analyzed showed minimal cross-reactivity with related compounds at the physiological concentrations found in the media. We will continue to compare the ELISA assays with LC-MS/MS, but, in this work, we are confident in the methods of analysis.
2. **Each immunoassay has a measuring range and a LLOQ. Are all data points included in the manuscript for all lipid mediators within the measuring range of the respective immunoassay? Response:** Indeed, all reported values for lipids in the manuscript exceed the LLOQ. We were very careful about this assertion.
3. **While expression analyses have been done for most enzymes necessary for lipid mediator production investigated in endothelial cells, the expression of ALOX15, necessary for LXA4 production is not shown in the manuscript. Response:** In the revised manuscript, we have included qRT-PCR and western blots showing expression of 15-LOX-1, including the use of THP-1 and HL-60 cell extracts as positive controls.
4. **What levels of lipid mediators are present in the medium containing FCS used to incubate the HUVECs? Response:** For all experiments, 0.5% charcoal-stripped FBS was used. We measured the levels of each lipid mediators in the 0.5% FCS and they are at or below the LLOQ for each ELISA.
5. **mPGES-1 protein expression is not visible on the western blot in Fig. 1H. Response:** In the revised manuscript, we have included a better western blot image to illustrate the expression of mPGES-1, the enzyme that synthesizes PGE_2_.
6. **The results of the qPCR analysis are shown as fold increase only. However, it would be interesting to see how low the baseline levels for the different mRNAs are. Response:** For the transcript expression experiments, we used the well-accepted 2^-ΔΔ^*^Ct^* method of Schmittgen & Livak (*Nature Protocols*, 2008; 3: 1101). This method takes into account the change in target gene relative to the expression of a reference gene based upon the relative *Ct* values of each transcript. However, Table 1 in the original submission also includes the absolute *Ct* values for ALOX5, LTA4H and FLAP. In the revised manuscript, we also include the *Ct* values for the other transcripts were measured.
7. **There are no figure legends included in the manuscript, which makes the interpretation of the figures hard. Response:** We sincerely apologize for this oversight. Somehow, the figure legend was not uploaded in the original submission. We have included the figure legend in the revised manuscript.
8. **It is not clear whether COX-1 and COX-2 are depicted in Figure 2A. Response:** Apologies for the lack of clarity. The y-axis for COX-2 is on the left (with blue type) and the y-axis for COX-1 is on the right (with red type) of Figure 2A. We used color coding of COX-2 and COX-1 on the same graph to illustrate the inducibility of COX-2 and the lower level, constituent expression of COX-1.
9. **The western blot depicted in figures 4 A) and B) do not support the results depicted in the respective graphs. Response:** We have prepared new western blots and densitometric analysis and have inserted these new data into Figure 4.
10. **Second last line on page 9 should be written Fig. 1C and not Fig. 1B. Response:** We thank the reviewers for pointing out this error. We have made the correction in the text.
11. **Figure 8 is not readable due to a too low resolution of the TIF files. Response:** These imaging glitches have been corrected.

**Reviewer #2: … a few grammar should be checked. Response:** Done.

1. **Please discuss the role of aspirin in low and high dose regarding anti-inflammatory and anti-thrombotic actions.** We made a few comments about the use of low-dose aspirin for preeclampsia versus the of aspirin as anti-platelet therapy.
2. **In the results section pleased avoid to discuss your finding or to cite bibliography or to described the design of the study. Response:** The only instance in which we cited previous publications in the Results section (see **under IL-1b stimulates LTB4 synthesis in endothelial cells independent of leukocytes: Modulation by aspirin**) is relevant and supports the conduct of the experiment and has been retained in the revised manuscript.
3. **The conclusion should be shorten. Response:** Per the reviewer’s suggestion, we have shortened the Conclusions and Future Directions section.

**Reviewer #3:**

1. **As the authors point out, 5-LOX is typically not highly expressed in endothelial cells, although some evidence suggests that it can be induced under certain conditions (e.g., cytomegalovirus infection, hypoxia, etc). Response:** In the revised manuscript, we have included positive control THP-1 (monocyte/macrophage) and HL-60 (neutrophil) cell lysates for lipoxygenase western blots.
2. **The results show that native (15S) lipoxin A4 is produced in the HUVEC, impression of 15-LOX in this particular lot of cells (pooled donors I presume). Have the authors evaluated expression of 15-LOX type 1 or 2 in these cells? Response:** In the revised manuscript, we include western blots of 15-LOX-1 and 15-LOX-2 expression in HUVEC. It is worthy noting that the HUVEC cultures were from pooled donors and several separate batches of cells were used in the experiments.
3. **The inhibitor used, NDGA can be non-specific, inhibit several lipoxygenases, and potentially anti-oxidant/free radical scavenging effects. Formation of non-enzymatic lipid oxidation products could occur in these cells under inflammatory conditions and structurally similar non-leukotrienes for example (see point 4 below). Thus, a more specific approach like siRNA (as described in Point 1) or a distinct inhibitor should additionally be used to definitively document the enzymatic origin of leukotrienes and lipoxins in these cells. Response:** In the revised manuscript, we have included Zileuton, a specific 5-LOX inhibitor and MK-886, a FLAP inhibitor, in addition to NDGA, to document the specific LOX pathways involved in the production of leukotrienes and lipoxins in endothelial cells.
4. **(Related to Point 3): ELISAs for lipid mediators are known to have cross reactivity. For instance, the kits for LXA4 and 15R-LXA4 show cross reactivity for resolvin D1 and 17R-resolvin D1, respectively, while the kit for LTB4 shows cross reactivity for both 5S-HETE and 5R-HETE (which could be formed during oxidative stress non-enzymatically as a racemic mixture). As such, since a more specific approach such as LC-MS/MS is not being used, the authors should at least more thoroughly establish the enzymatic origin of these mediators using additional distinct and selective inhibitors of 5-LOX in cells co-treated with ASA for 15R-LXA4 and inhibitors of FLAP, LTA4H, and a distinct inhibitor of 5-LOX (other than NDGA) for LTB4, as noted above. Response:** We fully acknowledge that LC-MS/MS is the gold standard for accurate structural identification of lipids (and other biomolecules, for that matter, and that no antibody-based method is perfect). We provided our rationale for the use of ELISA rather than mass spectrometry in the responses listed above. In the revised manuscript, we have included the use of additional “specific” inhibitors of the LOX pathway (in the presence and absence of aspirin) to probe the LXA_4_ and 15R-LXA_4_ formation. Regarding the cross-reactivity of LXA_4_ and 15R-LXA_4_ ELISA kits with D-series resolvins, we observed NO evidence of resolving synthesis of the D-series in the absence of supplemental DHA. In the experiments described in the manuscript, we focused only on arachidonic acid-derived lipoxins (LXA_4_ and 15R-LXA_4_) using supplemental AA in the experiments.
5. **There are no figure legends are present in the manuscript and they should be included in a revision. Response:** We apologize for the oversight in the original submission and have included a detailed figure legend in the revision.
